# Identification of Natural Hybrids between *Ahlbergia frivaldszkyi* (Lederer, 1853) and *Callophrys rubi* (Linnaeus, 1758) (Lepidoptera, Lycaenidae) Using Mitochondrial and Nuclear Markers

**DOI:** 10.3390/insects12121124

**Published:** 2021-12-15

**Authors:** Nazar A. Shapoval, Roman V. Yakovlev, Galina N. Kuftina, Vladimir A. Lukhtanov, Svyatoslav A. Knyazev, Anna E. Romanovich, Anatoly V. Krupitsky

**Affiliations:** 1 Department of Karyosystematics, Zoological Institute, Russian Academy of Sciences, Universitetskaya Nab. 1, 199034 Saint-Petersburg, Russia; galinakuftina@mail.ru (G.N.K.); lukhtanov@mail.ru (V.A.L.); 2 Institute of Biology, Tomsk State University, Lenina Pr. 36, 634050 Tomsk, Russia; yakovlev_asu@mail.ru; 3 Department of Ecology, Altai State University, Lenina Pr. 61, 656049 Barnaul, Russia; konungomsk@yandex.ru; 4 Resource Center for Development of Molecular and Cellular Technologies, Saint-Petersburg State University, Universitetskaya Nab., 7/9, 199034 Saint-Petersburg, Russia; aromanovich@gmail.com; 5 Department of Entomology, Biological Faculty, Lomonosov Moscow State University, Leninskie Gory, GSP-1, Korp. 12, 119991 Moscow, Russia; 6 Severtsov Institute of Ecology and Evolution, Russian Academy of Sciences, Leninsky Pr. 33, 119071 Moscow, Russia

**Keywords:** elfin butterflies, interspecific hybridization, intergeneric hybrids

## Abstract

**Simple Summary:**

Butterfly specimens with unusual morphological characters (e.g., unusual wing coloration) have contradictory interpretations in the literature and have been considered by different authors either as previously undescribed taxa, putative hybrids, or aberrations of well-known species. Such individuals clearly represent a taxonomic problem that needs to be addressed by scientists. The application of molecular techniques could shed light on the origin of morphological uncertainty. Here we use a combination of mitochondrial and nuclear DNA markers to analyze three lycaenid butterflies with unusual wing pattern, which are thought to represent naturally occurring hybrids due to their intermediate phenotype. We confirm their hybrid origin and indicate that the specimens are wild-caught hybrids between females of *Callophrys rubi* and males of *Ahlbergia frivaldszkyi*. Our data indicate that gene flow across species boundaries in these butterflies can occur long after speciation.

**Abstract:**

Natural hybridization is rather widespread and common in animals and can have important evolutionary consequences. In terms of taxonomy, exploring hybridization and introgression is crucial in defining species boundaries and testing taxonomic hypotheses. In the present paper, we report on natural hybrid specimens between *Ahlbergia frivaldszkyi* (Lederer, 1853) and *Callophrys rubi* (Linnaeus, 1758). To test the hypothesis of their hybrid origin, we employed the molecular mitochondrial (*COI* gene) and nuclear (*wingless*, *RPS5*, and *Ca-ATPase* genes) markers commonly used in phylogenetic studies and explored the morphology of the specimens. Our analysis revealed that hybrids bear mitochondrial haplotypes of *C. rubi*, while nuclear fragments are heterozygous, sharing a combination of *A. frivaldszkyi* and *C. rubi* lineages. The hybrid specimens combine morphological characters of both genera. Our results for the first time empirically demonstrate the possibility of genetic introgression between these species and between the genera *Callophrys* and *Ahlbergia* on the whole.

## 1. Introduction

Individuals with exceptional phenotypic traits (e.g., unusual coloration or deviant morphological characters) clearly represent a taxonomic issue that needs to be addressed by scientists. Such individuals are often considered either as previously undescribed taxa or aberrations of well-known species, but could also be putative hybrids, i.e., represent consequences of hybridization.

Hybridization is defined as the reproduction between members of genetically distinct populations [1] producing offspring of mixed ancestry. Although natural hybridization is usually rare on a per-individual level, appearing of natural hybrids indicates that introgression between species, i.e., invasion of foreign genetic material into a native genome [2], is an ongoing and regular process in nature. On a per-species basis, hybridization events are rather widespread and common in animals [2,3] and can have important evolutionary consequences. In particular, hybridization may lead to speciation when two species hybridize and give rise to a novel independent species [2,3,4]. In terms of taxonomy, exploring hybridization and introgression is crucial in defining species boundaries [5] and testing existing taxonomic hypotheses.

Incorporation of new tools and techniques, such as analysis of mitochondrial (mtDNA) and nuclear (nucDNA) sequence data [6], amplified fragment length polymorphism (AFLP) [7], genome-wide genotypic [8] and inter-simple sequence repeat (ISSR) markers [9,10], allow testing phenotypically deviant individuals more precisely and clearly detect the hybrid origin of organisms. Modern data show that hybridization and introgression are more common and widespread events than it was assumed previously [11].

Hybridization is reported for different groups of butterflies, including lycaenids (Lepidoptera, Lycaenidae) (e.g., [10,11,12,13,14]), and may occur even between distinct genera or species that bear drastically different karyotypes, i.e., chromosome numbers [4,15,16,17]. In particular, intermediate specimens tentatively characterized as natural intergeneric/intersubgeneric hybrids were found by Warren and Robbins [15] (the hybrid between *Callophrys* (*Callophrys*) *sheridanii* (Edwards, 1877) and *Callophrys* (*Incisalia*) *augustinus* (Westwood, 1852)) and Ivonin and co-authors [17] (the hybrid between *Ahlbergia frivaldszkyi* (Lederer, 1853) and *Callophrys rubi* (Linnaeus, 1758)), though conclusions about their hybrid nature were not confirmed by molecular phylogenetic analyses.

*Callophrys* Billberg, 1820 (sensu stricto) is a Holarctic genus of the tribe Eumaeini, subfamily Theclinae, comprising about 30 species distributed in Eurasia and North America [18,19,20,21,22,23,24]. *Ahlbergia* Bryk, 1947 is a genus of the tribe Eumaeini, subfamily Theclinae, occurring in mountains of East Asia, with a peak number of species in China [25,26]. A consistent survey of *Ahlbergia* was started relatively recently by Johnson [25], who outlined three so-called “Palaearctic elfin butterflies” genera, namely, *Ahlbergia*, *Cissatsuma* Johnson, 1992, and *Novosatsuma* Johnson, 1992, differing in genitalia structure. Since Johnson’s study, the total number of the *Ahlbergia* species has been raised to 33 [27,28,29].

The taxonomy of *Ahlbergia* and *Callophrys* is still debatable. Gillham [30] recognized *Ahlbergia* as a synonym of the New World genus *Incisalia* Scudder, 1872 based on the analysis of genitalia structure. Robbins 2004 [31] considered all the genera of the Palaearctic elfin butterflies sensu Johnson [25] and the American elfin genera *Incisalia*, *Mitoura* Scudder, 1872, *Sandia* Clench & Ehrlich, 1960, *Xamia* Clench, 1961, *Cisincisalia* Johnson, 1992, *Loranthomitoura* Ballmer & Pratt, 1992 and *Deciduphagus* Johnson, 1992 synonyms of *Callophrys*. Opler and Warren (2002) [32] and Pelham [33] treated some of these taxa as subgenera of *Callophrys* sensu lato; Pratt and co-authors [18] considered some of these taxa separate genera. Gorbunov [34] and Gorbunov and Kosterin [35] treated *Ahlbergia* as a subgenus of *Callophrys*. Ten Hagen and Miller [19] came to the same conclusion based on the phylogenetic analysis of the barcoding region of the mitochondrial *cytochrome C oxidase subunit I* gene (*COI*).

*Callophrys rubi* and *A. frivaldszkyi* are the most common and widespread species of the genera *Callophrys* and *Ahlbergia*, respectively. The former species is distributed in entire Europe, North Africa, Turkey, northeast Iran, Near East, Siberia, and mountains of Central Asia [19]. The distribution range of the latter species covers a large part of Siberia from the Ural, Altai and Sayan Mountains to the continental Far East of Russia and Sakhalin, Korean Peninsula, Eastern and Central China (provinces of Liaoning, Heilongjiang, Beijing, Tianjin, Hebei, Shanxi, Shaanxi, Gansu, Chongqing, and Guizhou) [27].

*Ahlbergia frivaldszkyi* and *C. rubi* broadly share their range and habitats in Siberia. An intermediate specimen sharing external characters of both species was reported from Novosibirsk Oblast, Russia, by Ivonin and co-authors [17]. The authors considered it as a putative hybrid between these species. This assumption was made on the basis of analysis of external morphology alone and was not confirmed by molecular methods or analysis of genitalia structure.

**Figure 1:**
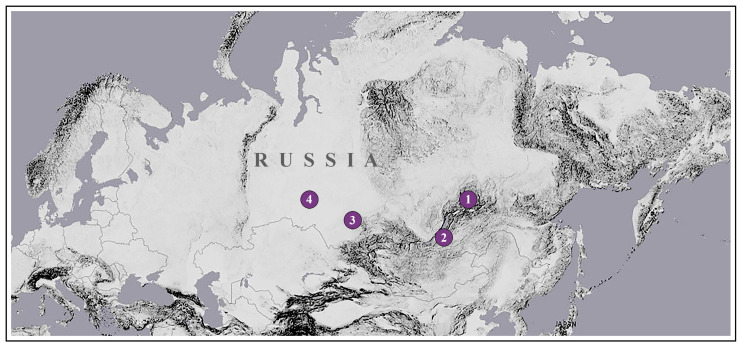
Map showing sampling localities of the analyzed specimens of putative hybrids and parental species. (1) Russia, Irkutsk Oblast, vic. Bodaybo; (2) Russia, Buryatia Republic, vic. Ulan-Ude city; (3) Russia, Novosibirsk Oblast, Matveevsky Range, Poldnevaya riv.; (4) Russia, Omsk Oblast, vic. Samsonovo vill.

At the beginning of June 2016, a putative hybrid specimen sharing external characters of *A. frivaldszkyi* and *C. rubi* was collected by Roman Yakovlev in the vicinity of Bodaybo town (Irkutsk Oblast, Russia). Other specimens possessing phenotype intermediate between *A. frivaldszkyi* and *C. rubi* were collected by Anatoly Filippov in mid-May 2018 in the vicinity of Ulan-Ude city (Russia, Buryatia Republic) and by Svyatoslav Knyazev in May 2021 in the vicinity of Samsonovo village (Russia, Omsk Oblast) (Figure 1).

In this study, we describe the morphology of the specimens in question and employ the molecular mitochondrial and nuclear markers commonly used in phylogenetic studies to test the hypothesis of the hybrid origin of these specimens and, in the event our hypothesis is correct, to identify the direction of the introgression. Additionally, we compare the external morphology of these specimens with a specimen reported by Ivonin and co-authors [17] from Novosibirsk Oblast.

## 2. Materials and Methods

### 2.1. Specimens Sampling

Putative hybrids and parental specimens were collected in Irkutsk and Omsk Oblasts (Russia) by R.V.Y. and S.A.K. during the field studies in 2016 and 2021, respectively. Searches for putative hybrid specimens in other sites of *C. rubi* and *A. frivaldszkyi* co-occurrence (conducted at “Aktru” Research Station, Altai Krai, Kosh-Agatch district, 50°03′37.1″ N; 87°24′05.0″ E) were unsuccessful. Legs of another putative hybrid specimen collected in the vicinity of Ulan-Ude city (Buryatia, Russia), as well as *A. frivaldszkyi* and *C. rubi* specimens collected sympatrically and synchronously with the latter, were kindly provided by Anatoly Filippov (Russia, Ulan-Ude). Photos of the putative hybrid specimen reported by Ivonin and co-authors [17] from Novosibirsk Oblast were used for comparison. The list of the specimens used for the molecular analysis and the full collection data are given in Table 1. Collection acronyms used throughout the text are as follows: AFU, collection of Anatoly Filippov, Russia, Ulan-Ude; RYaB, collection of Roman Yakovlev, Russia, Barnaul; SKO, collection of Svyatoslav Knyazev, Russia, Omsk; SZMN, Siberian Zoological Museum, Novosibirsk, Russia.

### 2.2. Molecular Markers, DNA Extraction, and PCR Amplification

One mitochondrial (cytochrome oxidase subunit I (*COI*) gene) and three nuclear (ribosomal protein S5 (*RpS5*), wingless (*Wg*), and sarco/endoplasmic reticulum calcium ATPase (*Ca-ATPase*)) genes were used as molecular markers.

One leg from each specimen was taken for DNA extraction using QIAamp DNA Investigator Kit (Qiagen, Venlo, The Netherlands) following the manufacturer’s protocol. Mitochondrial DNA barcode (a 658 bp fragment of the *COI* gene) was amplified using LCO1490/HC2198 primer pair [36]. Primers HybLepWG1/HybLepWG2 [37], HybrpS5degF/HybrpS5degR [38], Ca-ATPase_F/Ca-ATPase_R [39] were used for nucDNA amplification and resulted in 403 bp fragment of the *Wingless*, 610 bp fragment of *RPS5*, and 445 bp fragment of *Ca-ATPase* genes, respectively.

The PCR amplifications were performed in a 25 μL reaction volume per sample. Each reaction contained 1 μL template DNA (ca. 10–50 ng genomic DNA), 1.3 μL of both forward and reverse primers aliquoted to a standard concentration of 10 μM, 5 μL of 5× ScreenMix (Evrogen, Moscow, Russia), and 16.4 μL of ddH_2_O. The temperature profile for *COI, RPS5*, *Ca-ATPase*, and *Wingless* genes was as follows: initial denaturation at 95 °C for 5 min, followed by 35 cycles of denaturation at 94 °C for 30 s, annealing at 50 °C (*COI*, *Wingless*)/55 °C (*RPS5*, *Ca-ATPase*) for 30 s, and extension at 72 °C for 1 min 30 s, with a final extension at 72 °C for 10 min. The purified PCR products were subjected to the further sequencing. We cloned *Wingless* and *Ca-ATPase* genes for three putative hybrid specimens for which standard sequencing revealed intra-individual heterogeneities in the form of single nucleotide polymorphism. The cloning procedure was performed following previously described protocols [4,40]; 10 clones of each gene per specimen were sequenced. Sequencing of the double-stranded product was carried out at the Research Resource Center for Molecular and Cell Technologies (St. Petersburg State University, St. Petersburg, Russia) using ABI 3500xL analyzer (Applied Biosystems, Waltham, MA, USA).

### 2.3. Phylogenetic Reconstructions

The sequences were checked, edited and aligned using CHROMAS 2.6.6 (http://www.technelysium.com.au/ (accessed on 20 September 2021)) and Geneious Prime 2021.2.2 [41] software. Primer sequences were cropped. Heterogeneous nucleotide positions of nuclear genes were identified through dual peaks present in electropherograms and coded as degenerate base symbols. Three nuclear genes were concatenated resulting in the final data set comprising a total of 1458 bp (403 bp of the *Wingless*, 610 bp of the *RpS5*, and 445 bp of the *Ca-ATPase*). The mitochondrial *COI* gene was analyzed separately. A Bayesian approach was used to estimate the phylogeny. The analyses were performed using the MrBayes v3.2.7a software [42] with the nucleotide substitution model GTR + G + I as suggested by jModelTest v2.1.10 [43]. Two independent MCMC runs of 10 million generations, with four simultaneous chains (one cold and three heated) for each analysis, were performed. The sampling of trees and parameters was set to every 1000 generations. The first 10% of trees were discarded as burn-in prior to computing a consensus phylogeny and posterior probabilities. The consensus of the obtained trees was visualized using FigTree v1.4.4 (http://tree.bio.ed.ac.uk/software/figtree/, (accessed on 20 September 2021). TRACER, v1.7.1 was used for summarizing the results of the Bayesian phylogenetic analysis (http://beast.bio.ed.ac.uk/Tracer (accessed on 20 September 2021).

Since similar fragments of mitochondrial and three nuclear genes were obtained by us previously for *Colias palaeno* (Linnaeus, 1761), we used *COI* sequence and *Wingless* + *RpS5* + *Ca-ATPase* concatenated sequence of one *C. palaeno* specimen to root the mitochondrial and nuclear phylograms.

### 2.4. Morphological Analysis

The nomenclature of the genitalia and wing pattern is adapted after Johnson [17] and [44]. The nervuration nomenclature follows Comstock-Needham system adapted for butterflies [45]. Abdomens of the studied specimens were removed and macerated in 10% KOH for the examination of the male genitalia. After cleaning in water and dehydration in 96% EtOH, a genital capsule with valvae and separated aedeagus were placed in a drop of glycerol, covered with a cover glass and, photographed. In the case of the genital capsule, photos were taken in ventral and lateral views, and in lateral view in the case of the aedeagus. The images of the studied specimens were taken with a digital camera Canon EOS 5D mark II (Canon Inc., Tokyo, Japan) equipped with Sigma 150 mm f2.8 lens (Sigma Corporation, Kawasaki, Japan), using originally developed light system and a flash Canon Speedlight 430 EX (Canon Inc., Tokyo, Japan) with a diffuser. The images of the genitalia were taken with a Canon EOS 6D digital camera (Canon Inc., Tokyo, Japan) equipped with a Canon MP-E 65 mm f/2.8 lens (Micromed, St. Petersburg, Russia), using two Micromed Dual Goose illuminators. Obtained images were edited using Adobe Photoshop CC 2014.2.2 software.

## 3. Results

### 3.1. Morphology

#### 3.1.1. Ahlbergia frivaldszkyi

**Figure 2:**
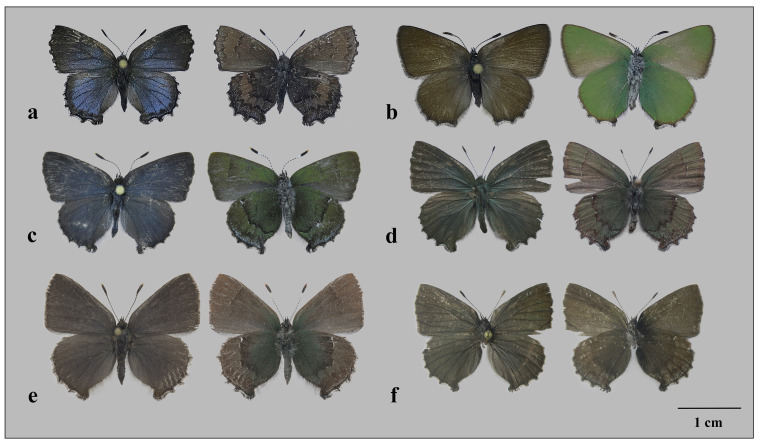
Putative hybrid specimens and parental species, dorsal (left) and ventral (right) side of wings (**a**–**f**). (**a**) *A. frivaldszkyi*, (**b**) *C. rubi*, (**c**) *A. frivaldszkyi* × *C. rubi*, sample ID—CFR01 (Russia, Irkutsk Oblast, vicinity of Bodaybo town, 01–02.VI.2016, R.V. Yakovlev leg. (RYaB)), (**d**) *A. frivaldszkyi* × *C. rubi*, sample ID—CFR02 [Russia, Buryatia Republic, Pribaikalskij district, 10 km N of Ulan-Ude city, 51°57′15.92″ N; 107°41′23.62″ E, 15.V.2018, A.V. Filippov leg. (AFU)], photo A. Filippov, (**e**) *A. frivaldszkyi* × *C. rubi*, sample ID—CFR03 (Russia, Omsk Oblast, Tarskiy district, 3 km E of Samsonovo vill., 56°58′27.90″ N; 74°24′43.39″ E, 15.V.2021, S.A. Knyazev leg. (SKO)), (**f**) *A. frivaldszkyi* × *C. rubi* hybrid, (Russia, Novosibirsk Oblast, Maslyaninskiy district, Matveevsky Range, Poldnevaya riv., 14.V.2004, Nikolaev leg. (SZMN)), photo O. Kosterin.

**Male** (Figure 2a).

**Head:** antenna black, white-ringed at base of segments, club dark, its base white ventrally, apiculus brown. Eye dark brown with small pale brown hairs, surrounded with white scales. Frons grey-brown with dark brown hairs, top of head with tuft of dark brown and whitish hairs.

**Palpus:** 2nd and 3rd segment dark brown with admixture of white scales and dark brown hairs.

**Thorax:** dorsal side dark grey with dark blue-grey hairs; ventral side with whitish hairs. Leg grey with white rings at base of tarsomeres.

**Abdomen:** dark brown dorsally, ventral side with whitish hairs.

**Forewing:** triangular with somewhat wavy margin and rounded apex. Forewing length usually 8.0–15.0 mm. Dorsal side of forewing brown with bluish shine and blue scales, more intensive basally. Veins dark brown. Margin dark brown proximally, light brown distally. Fringe dark brown proximally, whitish distally, veins marked by dark brown tufts. Androconial spot oblong, very narrow, brown, length about 1.0 mm. Ventral side of forewing generally brown, dark brown basally, light brown in spaces CuA2–2A. Postmedial line usually well-developed, dark brown from inside, suffused with whitish scales from outside. Crescent line usually poorly developed, submarginal area suffused with scattered white scales. Margin and fringe as on dorsal side.

**Hindwing:** rounded, with straight costal edge, crenated margin and well-developed anal lobe. Dorsal side brown with more or less developed blue field interrupted by brown spots in submarginal area of spaces M3–CuA2. Margin dark brown proximally, light brown distally. Fringe dark brown proximally, whitish distally, veins marked by dark brown tufts; anal lobe with dark brown scales and hairs. Ventral side of wing motley, covered with scattered white scales. Basal disc dark brown, its marginal band wavy, of irregular shape, with large projection in space M3 typical for the genus. Postbasal marks usually indistinct. Outer margin of band of disc covered with white scales, usually more intensively in spaces Sc + R1 and 2A–3A. Crescent line dark brown, limbal area covered with scattered white scales. Margin, fringe, and anal lobe as on dorsal side.

**Figure 3:**
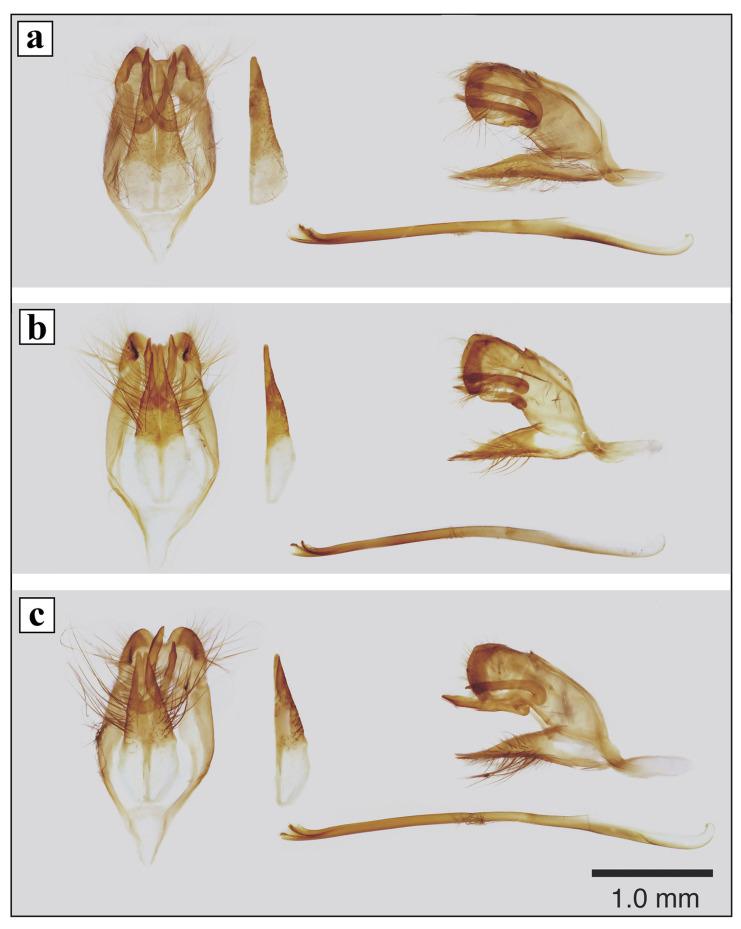
Male genitalia of the parental species and a putative hybrid (**a**–**c**): genital capsule with valvae, ventral view; right valva, ventral view; genital capsule with valvae, lateral view; aedeagus, lateral view. (**a**) *A. frivaldszkyi*, sample ID—10FR, (Russia, Irkutsk Oblast, vicinity of Bodaybo town, 01–02.VI.2016, R.V. Yakovlev leg. (RYaB)), (**b**) *C. rubi*, sample ID—05RUB, (Russia, Irkutsk Oblast, vicinity of Bodaybo town, 01–02.VI.2016, R.V. Yakovlev leg. (RYaB)), (**c**) *A. frivaldszkyi* × *C. rubi* putative hybrid, specimen ID—CFR01 (Russia, Irkutsk Oblast, vicinity of Bodaybo town, 01–02.VI.2016, R.V. Yakovlev leg. (RYaB)).

**Male genitalia** (Figure 3a): annulus somewhat wider than uncus, extended in middle part of genital capsule; lobes of uncus with well-developed chitinous processes rounded at tips; falx stout, pointed; valva broad, lanceolate, strongly broadened basally, gradually tapering to apex, tip of valva rounded; saccus rather short, triangular; aedeagus rather long, about 1.6x genitalia length, slightly curved, with straight upper cornutus and arcuate lower cornutus.

**Female.**

Similar to male, blue fields on both wings usually wider and of more intensive color.

#### 3.1.2. Calloprhys rubi

**Male.**

**Head:** antenna black, white-ringed at base of segments, club dark, its base white below, apiculus brown. Eye brown with small pale brown hairs, surrounded with green scales. Frons dark brown with pale hairs, top of head with brownish hairs.

**Palpus:** 2nd segment greenish-white with green scales and white, black and pale green hairs below; 3rd segment black with white hairs and scales.

**Thorax:** dorsal side brown with grey hairs, ventral side with whitish hairs. Leg white with brown scales and white hairs and with white rings at base of tarsomeres.

**Abdomen:** brown dorsally, ventral side with whitish hairs.

**Forewing:** triangular with rounded apex. Forewing length usually 10.0–15.0 mm. Dorsal side of forewing brown. Veins and margin dark brown. Fringe brown proximally, whitish distally. Androconial spot ovoid, light or brown, length about 1.0 mm. Ventral side of forewing grassy green, with emerald green scales near base of wing, light brown in spaces CuA2–2A. White postmedial line usually absent, rarely developed. Margin brown proximally, light brown distally. Fringe as on dorsal side.

**Hindwing:** rounded, with somewhat wavy margin and well-developed anal lobe. Dorsal side of forewing brown. Veins dark brown. Margin dark brown. Fringe brown proximally, whitish distally, veins marked by brown tufts. Anal lobe marked with dark brown scales and hairs. Wing densely covered with long brown or ochraceous hairs, more intensively at base and anal lobe. Ventral side of hindwing grassy green, with emerald green scales near base of wing. White postmedial line consisted of white strikes suffused with brownish scales from inside, rarely fully developed, usually strongly reduced to single strike in space Sc + R1 or totally absent. Margin, fringe, and anal lobe as on dorsal side.

**Male genitalia** (Figure 3b): annulus somewhat wider than uncus; lobes of uncus with well-developed chitinous processes rounded at tips; falx stout, pointed; valva narrow, slightly broadened in membranous part and narrowed at base, extended, evenly pointed to tip; saccus rather long, narrow, rounded at tip; aedeagus rather long, about 1.5× genitalia length, slightly curved, with straight upper cornutus and arcuate lower cornutus.

**Female** (Figure 2b).

Similar to male.

#### 3.1.3. Putative Hybrid Specimens *A. frivaldszkyi* × *C. rubi*

Specimen_ *A. frivaldszkyi* × *C. rubi* CFR01 (Figure 2c)

**Head:** antenna black, white-ringed at base of segments, club dark, its base white ventrally, apiculus brown. Eye dark brown with small pale brown hairs, surrounded with white scales. Frons grey-brown with dark grey hairs, top of head with tuft of dark grey hairs. Palpus: 2nd and 3rd segment dark grey with admixture of white scales and dark brown hairs. Thorax: upperside dark grey with dark blue-grey hairs; underside with grey hairs. Legs dark grey with black scales and white hairs.

**Abdomen:** dark grey dorsally, ventral side with grey hairs.

**Forewing**: triangular with straight margin between veins M3–A1–2A and rounded apex. Forewing length 13.0 mm. Dorsal side of forewing dark steel with bluish shine, more intensive basally, and rare scattered blue scales. Fringe dark brown proximally, grey distally, darker at veins. Androconial spot oblong, rather narrow, brownish grey, length about 1.0 mm. Ventrally forewing greyish-brown in spaces CuA2–2A, green from base of wing to transverse vein, greyish-brown with admixture of green scales in postdiscal area, dark brown blurred postmedial line and brown submarginal area. Margin greyish-brown, fringe as on dorsal side.

**Hindwing**: rounded, with wavy margin and well-developed anal lobe. Dorsal side steel with bluish shine and rare scattered blue scales. Margin dark brown. Fringe brown proximally, dirty white between veins and dark brown at veins distally; anal lobe with black and white scales and hairs. Ventral side of wing dirty green basally, green with admixture of brown and emerald scales in basal disc, postbasal marks absent, with group of dark scales marking transverse vein, rather broad dark marginal band of disc of irregular shape, U-curved at inner margin, marked with groups of white scales in middle part and at costa; postdiscal area green with emerald scales, emerald suffusion more intensive in spaces CuA2–2A near marginal band of disc; crescent line with blurred margin, dark brown with admixture of emerald scales; submarginal (limbal) area dark brown with intensive suffusion of white scales in spaces M1–CuA2; anal lobe with dark brown spot and long dark brown scales and hairs; margin dark brown; fringe as on dorsal side.

**Genitalia** (Figure 3c): annulus somewhat wider than uncus, extended in middle part of genital capsule; lobes of uncus with well-developed chitinous processes rounded at tips; falx stout, pointed; valva lanceolate, somewhat broadened basally, gradually tapering to apex, with nearly straight outer margin, tip of valva slightly bent to side; saccus long, triangular; aedeagus rather long, about 1.8× genitalia length, curved, with straight upper cornutus and arcuate lower cornutus.

Specimen *A. frivaldszkyi* × *C. rubi* CFR02 (Figure 2d)

Compared with the specimen CFR01, the specimen from Buryatia differs in dirty green ventral side of wings (green with admixture of emerald scales in specimen CFR01) as well as more serrated marginal band of disc (wavy marginal band of disc in specimen CFR01). Genitalia were not studied.

Specimen *A. frivaldszkyi* × *C. rubi* CFR03 (Figure 2e)

Rather worn specimen generally resembling the specimen CFR02. Differs from both previous specimens in less developed bluish dorsal coloration of wings as well as less developed ventral green fields of wings. Genitalia as in the specimen CFR01.

Specimen *A. frivaldszkyi* × *C. rubi* (Figure 2f)

A detailed description was given by Ivonin and co-authors [17]. Differs from the previous specimens in brown coloration of wings underside lacking green scales. Basal disc of hindwing dark brown, postdiscal area brown. Genitalia were not studied by us as the specimen was not accessible.

### 3.2. Phylogenetic Analysis

Analysis of the 658 bp fragment of the mitochondrial *COI* gene of *A. frivaldszkyi* demonstrated that specimens collected in Buryatia, Irkutsk, and Omsk share a unique single haplotype (**Ah01**) differing in at least four fixed nucleotide substitutions (overall mean p-distance is 0.4%, SE = 0.2%) from *C. rubi* specimens collected at the same localities (Table 2). On the contrary, analyzed specimens of *C. rubi* form three haplotypes: **Cl01**, common for Buryatia, Irkutsk and Omsk populations; **Cl02**, found only in a single specimen from Irkutsk and differing from the haplotype **Cl01** in one nucleotide substitution (A => G) in the position 310; **Cl03**, found in a single specimen from Buryatia and differing from the haplotype **Cl01** in three nucleotide substitutions: T => C in the positions 82 and 400, and C => A in the position 529.

The putative hybrid specimens from Irkutsk and Buryatia share their mitochondrial haplotypes (**Cl01** and **Cl03**, respectively) with *C. rubi,* while the third putative hybrid specimen from Omsk is characterized by the unique haplotype **AhCl01** differing from the most common for *C. rubi* haplotype **Cl01** in one nucleotide substitution T => C in the position 406.

**Figure 4:**
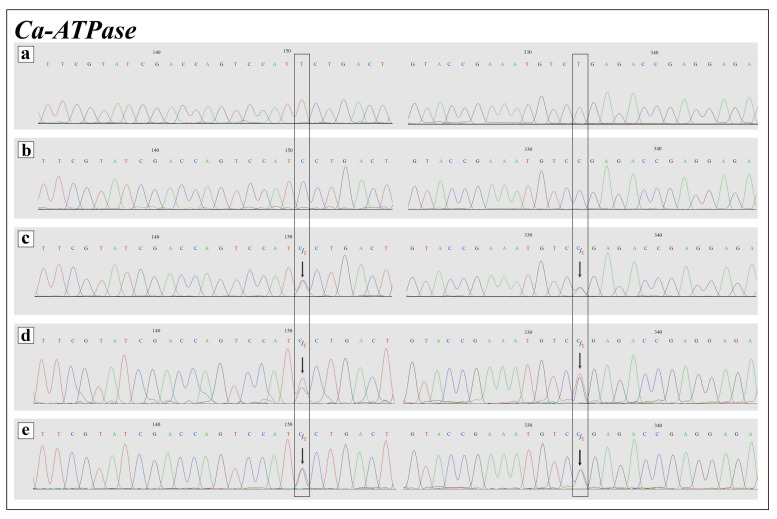
Electropherogram of *Ca-ATPase* nuclear gene fragment of parental species and putative hybrids (**a**–**e**). (**a**) *A. frivaldszkyi*, (**b**) *C. rubi*, (**c**) *A. frivaldszkyi* × *C. rubi*, sample ID—CFR01, (**d**) *A. frivaldszkyi* × *C. rubi*, sample ID—CFR02, (**e**) *A. frivaldszkyi* × *C. rubi*, sample ID—CFR03. Fixed *A. frivaldszkyi* × *C. rubi* interspecific nucleotide differences are boxed. Arrows indicate mixed signals in putative hybrids.

**Figure 5:**
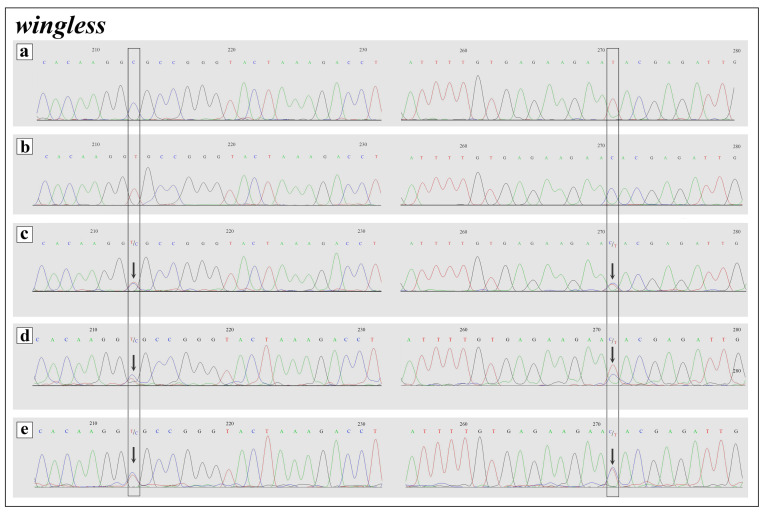
Electropherogram of *Wingless* nuclear gene fragment of parental species and putative hybrids **(a**–**e**). (**a**) *A. frivaldszkyi*, (**b**) *C. rubi*, (**c**) *A. frivaldszkyi* × *C. rubi*, sample ID—CFR01, (**d**) *A. frivaldszkyi* × *C. rubi*, sample ID—CFR02, (**e**) *A. frivaldszkyi* × *C. rubi*, sample ID—CFR03. Fixed *A. frivaldszkyi* × *C. rubi* interspecific nucleotide differences are boxed. Arrows indicate mixed signals in putative hybrids.

Analysis of the 610 bp fragment of the nuclear *RPS5* gene of the studied *A. frivaldszkyi* and *C. rubi* specimens reveals a low level of intraindividual heterozygosity (as evidenced by dual peaks of similar height in the electropherograms), and no fixed nucleotide substitutions separating these species. Analysis of the nuclear *Wingless* and *Ca-ATPase* genes fragments detect no intraindividual heterozygous sites in all of the studied *A. frivaldszkyi* and *C. rubi* specimens. At the same time, *A. frivaldszkyi* differs from *C. rubi* in two fixed substitutions of the studied *Wingless* (namely, positions 213 and 271) and *Ca-ATPase* (namely, positions 151 and 334) gene fragments. The fragments in question of the putative hybrid specimens are heterozygous in these positions sharing species-specific nucleotides of both, *A. frivaldszkyi* and *C. rubi* (Figure 4 and Figure 5).

**Figure 6:**
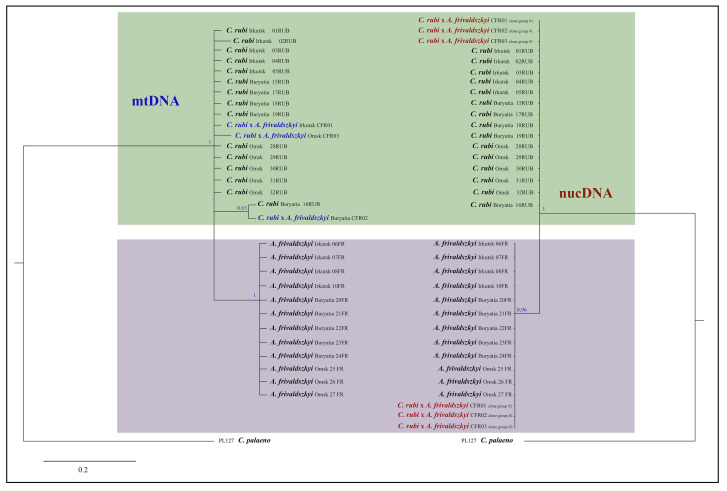
The Bayesian consensus trees of the analyzed specimens of *C. rubi*, *A. frivaldszkyi* and putative hybrids inferred from *COI* sequences (**left**) and concatenated alignment of three nuclear markers (*Wingless*, *RPS5*, *Ca-ATPase*) (**right**). Branches with Bayesian posterior probability values >0.60 are shown. Scale bar = 0.2 substitutions per position.

To confirm our hypothesis on a hybrid origin of specimens, we conducted a cloning procedure of the *wingless* and *Ca-ATPase* gene fragments of the putative hybrid specimens. This analysis revealed that 10 clones obtained for each gene fragment clearly split into two groups corresponding to *A*. *frivaldszkyi* variant (clone group #2) and *C. rubi* variant (clone group #1) (Figure 6). Thus, heterozygosity of the studied specimens revealed by direct sequencing of nuclear genes is a result of the shared combination of both lineages, *A*. *frivaldszkyi*-type and *C. rubi*-type. In the phylogenetic reconstructions based on the analysis of the 658 bp fragment of mitochondrial *COI* gene, *A*. *frivaldszkyi* and *C. rubi* were recovered together as a strongly supported monophyletic entity (PP = 1) (Figure 6). Within this entity, *A*. *frivaldszkyi* formed an independent well-supported lineage (PP = 1), whereas the specimens of *C. rubi* and the putative hybrids formed a paraphyletic cluster. One *C. rubi* specimen and the putative hybrid from Buryatia formed weakly supported clade (PP = 0.63), while the majority of the analyzed *C. rubi* specimens and the putative hybrid specimens from Irkutsk and Omsk Oblasts were placed on a polytomic branch opposed to the *A*. *frivaldszkyi* group. In the phylogenetic inference of the concatenated alignment of the nuclear markers, two groups of the sequenced hybrid clones, referred to the *A*. *frivaldszkyi*-type and *C. rubi*-type, were analyzed as independent samples (Figure 6). *Ahlbergia frivaldszkyi* and *C. rubi* specimens recovered together in the phylogenetic tree as a highly supported monophyletic entity (PP = 1). Within this entity, *A*. *frivaldszkyi*-type clones of putative hybrids and samples of *A*. *frivaldszkyi* formed a well-supported clade (PP = 0.96), whereas *C. rubi* specimens and *C. rubi*-type clones of the hybrid samples were found to be paraphyletic with respect to the *A*. *frivaldszkyi* lineage.

## 4. Discussion

The presence of the *COI* haplotypes of *C. rubi* and heterozygosity in fixed substitutions of analyzed regions of nuclear genes *wingless* and *Ca-ATPase* in the combination with intermediate morphological characters confirm our hypothesis on a hybrid origin of the specimens in question. In the cells of most animals, mtDNA is characterized by maternal inheritance, i.e., it is inherited solely from the mitochondria of the oocyte from which the animal develops [46]. In our case, the analysis revealed that the hybrid specimens inherited mtDNA from females of *C. rubi* and nuclear genes from both species.

An overall mean genetic distance of *COI* barcodes between analyzed specimens of *C. rubi* and *A*. *frivaldszkyi* is 0.4%. This value is much less than a species threshold of about 3%, which was empirically found for Lepidoptera [47]. Shared or very close *COI* mitochondrial barcodes in butterfly species, including lycaenids, can be explained by the mitochondrial introgression [4,48,49,50]. Ten Hagen and Miller [19] suggested the mitochondrial introgression in the genus *Callophrys* on the basis of an absence of fixed nucleotide substitutions in *COI* between morphologically differentiated species as well as the close genetic affinity between *C. rubi* and *A*. *frivaldszkyi*. Our results for the first time empirically demonstrate the possibility of genetic introgression between these species and between the genera *Callophrys* and *Ahlbergia* on the whole. The possibility of hybridization with further introgression of genome parts between these species is probably a result of similar genitalia structure, especially in males, and co-occurring in their habitats: both species are often spotted together [17]. Additionally, host plants of *A. frivaldszkyi*, *Spiraea* spp., are also known to be utilized by polyphagous *C. rubi* [51]. The cytogenetic background of the hybridization of *Callophrys* and *Ahlbergia* is unknown. Most Lycaenidae species have a haploid complement of either 23 or 24 chromosomes [52,53,54]. According to Federley [55] and Bigger [56], the haploid chromosome number of *C. rubi* is 23. The same number was reported for another Palaearctic species of the tribe Eumaeini, *Satyrium pruni* [51], so we cannot exclude the possibility that *Ahlbergia frivaldszkyi* also has a haploid complement of 23 chromosomes.

Externally, three revealed hybrid specimens from Irkutsk, Omsk Oblasts, and Buryatia combine characters of both genera. The following characters are of *A*. *frivaldszkyi*: forewing with angled outer margin (rounded in *C. rubi*), bluish tint of dorsal side of wings (wings brown dorsally in *C. rubi*), dark marginal band of disc (postdiscal band of white strikes in *C. rubi*), crescent line and suffusion of white scales of ventral side of hindwing (absent in *C. rubi*). Rounded hindwing (hindwing with straight costa and serrated outer margin in *A*. *frivaldszkyi*), green coloration of ventral side of wings, rounded marginal band of disc homologous to the postdiscal band of *Callophrys* (strongly incised marginal band of disc in *A*. *frivaldszkyi*) are the characters of *C. rubi.* An intermediate specimen between *C. rubi* and *A*. *frivaldszkyi* reported from Novosibirsk Oblast, which was considered a putative hybrid [17], lacks green coloration and more resembles *A*. *frivaldszkyi* than *C. rubi*.

In the male genitalia of the hybrid specimens, the shapes of annulus, uncus and saccus are generally of *C. rubi*, while the shape of valva is intermediate, with large slightly broadened basal portion as in *C. rubi* (shorter strongly broadened base of valva in *A*. *frivaldszkyi*) and nearly straight outer margin gradually tapering to apex as in *A*. *frivaldszkyi* (concave outer margin of valva in *C. rubi*).

Another known example of hybridization in the elfin butterflies resembling our case is a putative natural hybrid between Nearctic species *Callophrys* (*Callophrys*) *sheridanii* (Edwards, 1877) and *Callophrys* (*Mitoura*) *augustinus* (Westwood, 1852) described by Warren & Robbins [15]. This specimen combines green coloration of *C.* (*C.*) *sheridanii* and details of wings pattern of *C.* (*M.*) *augustinus*, while most of the characters (shape of hindwing, shape and position of hindwing marginal band of disc, shape of the genitalic sclerites) are intermediate. Our findings revealed homology of the marginal band of disc of *Ahlbergia* and the white postdiscal band of *Callophrys*, supporting the hypothesis of Warren & Robbins [15] of homology of these elements of the pattern in *Callophrys* and *Mitoura*. Interestingly, green coloration and developed pattern of the ventral side of the wings of the studied hybrid specimens from Irkutsk, Omsk Oblasts, and Buryatia resemble those of some Nearctic *Callophrys*, especially *C. gryneus* (Hübner, 1819).

From the taxonomic point of view, our findings demonstrate that *Callophrys* and *Ahlbergia* are very close genetically, but do not put a period to long-lasting argues if the *Callophrys* should be treated as a diverse genus uniting green hairstreaks and both elfin butterflies of Palaearctic and Nearctic as subgenera or Holarctic green hairstreaks and different groups of Holarctic elfin butterflies are separate genera. The final taxonomic conclusion should be based on a multilocus molecular phylogenetic analysis.

## 5. Conclusions

Our study shows that utilization of unlinked molecular marker analysis, namely mitochondrial and nuclear DNA genes, can successfully discriminate natural hybrids in the taxonomically complicated lycaenid genera *Callophrys* and *Ahlbergia*. The molecular findings are confirmed by intermediate morphological characters combining both traits of *C. rubi* and *A*. *frivaldszkyi* revealed in the hybrid specimens. The taxonomy of *Ahlbergia* and *Callophrys* is debatable, and the phylogenetic relationships of these genera still remain unclear. Our analyses demonstrate that *Callophrys* and *Ahlbergia* are very close genetically, and their phylogeny needs further investigations using a multilocus molecular phylogenetic analysis.

## Figures and Tables

**Table 1:** List of the studied materials.

Taxon	Sample ID	GenBank Accession Number				Locality
		*COI*	*Wingless*	*Ca-ATPase*	*RPS5*	
*Callophrys rubi* × *Ahlbergia frivaldszkyi*	CFR01	MW785873	MW811215 MW811216	MW811223 MW811224	MW811246	Irkutsk *
*Callophrys rubi* × *Ahlbergia frivaldszkyi*	CFR02	MW785872	OL584270 OL584273	MW811225 MW811226	OL584250	Buryatia **
*Callophrys rubi* × *Ahlbergia frivaldszkyi*	CFR03	OL457027	OL584271 OL584272	OL584293 OL584294	OL584251	Omsk ***
*Callophrys rubi*	01RUB	MW785853	MW811207	OL584295	MW811237	Irkutsk
*Callophrys rubi*	02RUB	MW785854	MW811208	MW811219	MW811238	Irkutsk
*Callophrys rubi*	03RUB	MW785855	MW811209	MW811218	MW811239	Irkutsk
*Callophrys rubi*	04RUB	MW785856	MW811210	MW811217	MW811240	Irkutsk
*Callophrys rubi*	05RUB	MW785857	OL584274	OL584296	MW811241	Irkutsk
*Callophrys rubi*	15RUB	MW785862	OL584275	MW811227	OL584252	Buryatia
*Callophrys rubi*	16RUB	MW785863	OL584276	MW811228	OL584253	Buryatia
*Callophrys rubi*	17RUB	MW785864	OL584277	MW811229	OL584254	Buryatia
*Callophrys rubi*	18RUB	MW785865	OL584278	MW811230	OL584255	Buryatia
*Callophrys rubi*	19RUB	MW785866	OL584279	MW811231	OL584256	Buryatia
*Callophrys rubi*	28RUB	OL457028	OL584280	OL584297	OL584257	Omsk
*Callophrys rubi*	29RUB	OL457029	OL584281	OL584298	OL584258	Omsk
*Callophrys rubi*	30RUB	OL457030	OL584282	OL584299	OL584259	Omsk
*Callophrys rubi*	31RUB	OL457031	OL584283	OL584300	OL584260	Omsk
*Callophrys rubi*	32RUB	OL457032	OL584284	OL584301	OL584261	Omsk
*Ahlbergia frivaldszkyi*	06FR	MW785858	MW811211	MW811220	MW811242	Irkutsk
*Ahlbergia frivaldszkyi*	07FR	MW785859	MW811212	MW811221	MW811243	Irkutsk
*Ahlbergia frivaldszkyi*	08FR	MW785860	MW811213	MW811222	MW811244	Irkutsk
*Ahlbergia frivaldszkyi*	10FR	MW785861	MW811214	OL584302	MW811245	Irkutsk
*Ahlbergia frivaldszkyi*	20FR	MW785867	OL584285	MW811232	OL584266	Buryatia
*Ahlbergia frivaldszkyi*	21FR	MW785868	OL584286	MW811234	OL584265	Buryatia
*Ahlbergia frivaldszkyi*	22FR	MW785869	OL584287	MW811236	OL584264	Buryatia
*Ahlbergia frivaldszkyi*	23FR	MW785870	OL584288	MW811235	OL584263	Buryatia
*Ahlbergia frivaldszkyi*	24FR	MW785871	OL584289	MW811233	OL584262	Buryatia
*Ahlbergia frivaldszkyi*	25FR	OL457024	OL584290	OL584303	OL584267	Omsk
*Ahlbergia frivaldszkyi*	26FR	OL457025	OL584291	OL584304	OL584268	Omsk
*Ahlbergia frivaldszkyi*	27FR	OL457026	OL584292	OL584305	OL584269	Omsk

*—Russia, Irkutsk Oblast, Bodaibinskiy district, vicinity of Bodaybo town, 01–02.VI.2016, R.V. Yakovlev leg. **—Russia, Buryatia Republic, Pribaikalskiy district, 10 km N of Ulan-Ude city, 51°57′15.92″ N; 107°41′23.62″ E, 15.V.2018, A.V. Filippov leg. ***—Russia, Omsk Oblast, Tarskiy district, 3 km E of Samsonovo vill., 56°58′27.90″ N; 74°24′43.39″ E, 15.V.2021, S.A. Knyazev leg.

**Table 2:** Variable sites of the studied *COI* gene fragment among the 30 samples sequenced.

Taxon/Haplotype	Nucleotide Position								
	40	82	103	271	310	361	400	406	529
*Ahlbergia frivaldszkyi/*Ah01	G	T	T	A	A	C	T	T	C
*Callophrys rubi/*Cl01	A	T	C	T	A	T	T	T	C
*Callophrys rubi/*Cl02	A	T	C	T	G	T	T	T	C
*Callophrys rubi/*Cl03	A	C	C	T	A	T	C	T	A
*Ahlbergia frivaldszkyi* × *Callophrys rubi/*AhCl01	A	T	C	T	A	T	T	C	C
